# General Anesthetic Conditions Induce Network Synchrony and Disrupt Sensory Processing in the Cortex

**DOI:** 10.3389/fncel.2016.00064

**Published:** 2016-04-14

**Authors:** Thomas Lissek, Horst A. Obenhaus, Désirée A. W. Ditzel, Takeharu Nagai, Atsushi Miyawaki, Rolf Sprengel, Mazahir T. Hasan

**Affiliations:** ^1^Department of Molecular Neurobiology, Max Planck Institute for Medical ResearchHeidelberg, Germany; ^2^Department of Neurobiology, Interdisciplinary Center for Neurosciences, University of HeidelbergHeidelberg, Germany; ^3^Max Planck Research Group at the Institute for Anatomy and Cell Biology, Heidelberg UniversityHeidelberg, Germany; ^4^Laboratory for Nanosystems Physiology, Hokkaido UniversityHokkaido, Japan; ^5^RIKEN-Brain Science Institute, Laboratory for Cell Function DynamicsSaitama, Japan; ^6^Molecular Neurobiology, Neurocure Cluster of Excellence, Charite-UniversitätsmedizinBerlin, Germany

**Keywords:** anesthetics, isoflurane, cerebral cortex, two-photon imaging, genetically encoded calcium indicators

## Abstract

General anesthetics are commonly used in animal models to study how sensory signals are represented in the brain. Here, we used two-photon (2P) calcium activity imaging with cellular resolution to investigate how neuronal activity in layer 2/3 of the mouse barrel cortex is modified under the influence of different concentrations of chemically distinct general anesthetics. Our results show that a high isoflurane dose induces synchrony in local neuronal networks and these cortical activity patterns closely resemble those observed in EEG recordings under deep anesthesia. Moreover, ketamine and urethane also induced similar activity patterns. While investigating the effects of deep isoflurane anesthesia on whisker and auditory evoked responses in the barrel cortex, we found that dedicated spatial regions for sensory signal processing become disrupted. We propose that our isoflurane-2P imaging paradigm can serve as an attractive model system to dissect cellular and molecular mechanisms that induce the anesthetic state, and it might also provide important insight into sleep-like brain states and consciousness.

## Introduction

Changes in brain activity, with up and down states (Constantinople and Bruno, 2011), and synchrony/desynchrony (Klimesch, 1996) dynamics, tend to correlate with various behavioral states (Poulet and Petersen, 2008), such as wakefulness, sleep, and locomotion. It is known that general anesthesia alters neuronal and neuroglial activity parameters (Heinke and Koelsch, 2005; Windels and Kiyatkin, 2006; Thrane et al., 2012) and brain states (Cimenser et al., 2011). In particular, there is a very close similarity of brain activity under anesthesia to sleep-like brain states (Tung and Mendelson, 2004; Van Dort et al., 2008; Bonhomme et al., 2011). It has been suggested that general anesthesia induced changes in neural activity might provide fundamental insight into the cellular and network mechanisms (Ishizawa, 2007) that shift brain states along the unconscious-conscious continuum (Alkire et al., 2008; Forman and Chin, 2008; Shin et al., 2013; Mashour, 2014; Barttfeld et al., 2015; Meyer, 2015).

Anesthetic agents act on different molecular targets (Solt and Forman, 2007; Nau, 2008; Sanders et al., 2008; Urban, 2008; Zeller et al., 2008), which in turn alter neuronal network activity. It is thought that isoflurane operates by increasing activity of γ-aminobutyric acid type A receptors (GABA_A_R) and potassium channels (Franks and Lieb, 1988; Rudolph and Antkowiak, 2004; Hemmings et al., 2005; Eikermann et al., 2011). Ketamine appears to inhibit N-methyl- D-aspartic acid (NMDA) receptor activity (Rudolph and Antkowiak, 2004; Hemmings et al., 2005) and addition of xylazine, a α_2_-adrenoreceptor agonist, to ketamine enhances its sedative effects (Green et al., 1981; Lu et al., 2008) and exerts anticonvulsive properties (Green et al., 1981). Finally, urethane (ethyl carbamate) appears to act on both inhibitory and excitatory molecular targets (Hara and Harris, 2002).

Electroencephalographical (EEG) studies suggest that burst suppression (BS), which is characterized by a quasiperiodical pattern of high voltage activity (bursts) and flat line (suppression) periods (Liley and Walsh, 2013), appears to be quite similar in subjects under deep general anesthesia (Brown et al., 2010) and human patients suffering from hypoxic–ischemic trauma and coma (Young, 2000). It was further suggested that brain wide synchronous states of recurrent activity play a key role, possibly to generate unconsciousness. However, multielectrode recordings of the human cortex at a mesoscopic scale suggest that BS can be asynchronous with regard to different cortical areas and there are both corticocortical and thalamocortical circuits that possibly interact with each other to generate unconsciousness (Lewis et al., 2013; Bojak et al., 2015). It is conceivable that anesthetics acting as GABAergic modulators are likely to exert their anesthetic effects differently on local cortical circuits, possibly because different neurons are decorated with a different collection of ion channels and receptors. It is quite intriguing that isoflurane (Hartikainen et al., 1995; Akrawi et al., 1996), sevoflurane (Jäntti et al., 1998), propofol (Akrawi et al., 1996; Huotari et al., 2004), thiopental (Akrawi et al., 1996), or etomidate (Akrawi et al., 1996) evoke bursts in various model organisms during deep anesthesia after presentation of a range of visual, auditory, tactile, and noxious stimuli. Moreover, anesthesia appears to disrupt sensory boundaries because in a previously reported study tone-evoked neural responses were detected in the visual cortex (Land et al., 2012). How neurons in microcircuits change their activity patterns under the influence of different general anesthetics is still not known.

To investigate the effects of general anesthesia on visually identifiable neurons in cortical microcircuits, we performed *in vivo* two-photon calcium (Ca^2+^) imaging using a genetically-encoded fluorescent Ca^2+^ indicator (GECI) YC2.60 (Nagai et al., 2004; Yamada et al., 2011). It is known that Ca^2+^ transients in neurons closely reflect neuronal spiking activity and that the fluorescence changes of GECIs can therefore serve as a proxy for neuronal activity (Hasan et al., 2004; Wallace et al., 2008; Lütcke et al., 2010). Our experimental 2PI-GECI paradigm can help to dissect the local and distributed cortical circuits and other brain circuits that generate unconsciousness by anesthetic treatments and it may also provide some insight into the cellular and molecular mechanisms underlying consciousness.

## Materials and methods

### Animal welfare

All experiments conformed to the animal welfare guidelines of the Max Planck Society. Efforts were made to minimize numbers of animals used and all experimental procedures were approved by the local authorities (Regierungspräsidium Karlsruhe).

### Plasmid construct and virus purification

The YC3.60 gene in the plasmid, pAAV-hSYN-YC3.60 (Shevtsova et al., 2005; Lütcke et al., 2010), was removed and replaced with the YC2.60 (Nagai et al., 2004) gene. We generated rAAV with hybrid serotype 2/1 as described previously (Dogbevia et al., 2015).

### rAAV injection and chronic window surgery

Surgical implantation of the cranial window was performed on 6–7 weeks old C57BL/6N mice. The animals were anesthetized with an intraperitoneal injection of ketamine (65 mg/kg body weight) and xylazine (14 mg/kg body weight). The surgical procedure was started only after animals no longer responded to tail or paw pinch. Throughout the surgery, animals were kept on a heating pad to prevent hypothermia. Eye cream (Bepanthen, Bayer) was applied to the eyes of mice to prevent dehydration. The animals were stabilized on a stereotactic frame (Kopf instruments). The skin above the skull was cut open with a surgical knife and the somatosensory cortex region was carefully marked. Subsequently, the skull was thinned with a dental drill and the cranial bone was carefully removed with a forceps and Ringer buffer was applied to moist the area. Bleeding that occurs during surgical procedures was stopped by applying small pieces of Tachosil. Using a micropipette, a small volume (200 nl) of virus (AAV-hSYN-YC2.60) was injected into the somatosensory cortex (AP −1,5; ML +3.0; DV −0.4, −0.3; all in mm from bregma) at a depth of 500 μm from the pia as described previously (Wallace et al., 2008; Lütcke et al., 2010). The open area of the cranial bone was covered with a circular glass (4 mm diameter) and sealed with dental cement. The dura mater was kept moist with Ringer buffer during the whole procedure. A head-plate was attached to the skull with superglue and additional dental cement was used to close the gaps between head-plate and skull. To avoid post-operative dehydration, mice were injected with physiological saline solution. To reduce moderate acute pain, buprenorphine (Buprenex, 0.1 μg/g body weight) was injected after the surgical procedure. Mice were placed on a heating-pad while recovering from surgery.

### Chronic *in vivo* two-photon calcium imaging

Calcium imaging was performed using a commercial two-photon laser-scanning microscope (LSM 510 META, Zeiss), equipped with a Ti:sapphire laser (Chameleon Ultra II, Coherent), a 40 × water immersion objective (Achroplan 40×/0.8 W IR, Zeiss) and multialkali photomultiplier tubes (R6357, Hamamatsu). Cells labeled with YC2.60 were excited at 850 nm or 870 nm, and emitted light from cyan fluorescent protein (CFP) and yellow fluorescent protein (YFP) was detected after passing through a 510 nm dichroic beam splitter and, subsequently, through a blue bandpass filter (CFP, BP 435–485, Zeiss) and a green long-pass filter (YFP, LP 515, Zeiss). Images were acquired at a frame rate of ~4 Hz, with a resolution of 128 × 128 pixels with the software provided by the microscope manufacturer. To revisit the same cells, we used blood vessel patterns and stereotactic coordinates. Furthermore, custom-built titanium headplates and the corresponding fixation devices ensured reproducible fixation of the animals under the microscope.

### Sensory stimulation

Deflection of all whiskers on the animal's right side was achieved using a short train of air-puffs (single puff duration = 130 ms, rate = 5 Hz, number of puffs per train = 5, pressure = 25 psi). The stimulation pattern was programmed into a Master-8 stimulus generator (A.M.P.I.) and air puffs were generated by a Picospritzer (General Valve). For sound-only trials, the air-pressure was reduced to 0 psi and the stimulator was directed away from the animal. Each trial was 74 s long and contained 3 air-puff trains at 5, 30, and 55 s. To avoid short-term adaptive changes in neuronal responses, the interval between trials varied between 30 s and 2 min. Routine visual checks ensured for efficient and reproducible whisker deflection from trial-to-trial. Image recording and whisker stimulation were time-locked by triggering the Master-8 through the microscope's image acquisition software.

### Anesthetic conditions for long-term functional imaging

Mice were anesthetized with isoflurane, ketamine alone, ketamine/xylazine or urethane. For detailed description of criteria for anesthetic depth, see Supplementary Table 1. Body temperature was monitored through a heat sensor beneath the animal and kept at 37°C with a heating blanket. Under deep anesthesia, lotion (Bepanthen) was applied to the eyes to prevent drying. In between trials, the animal's behavior was closely monitored and documented. For isoflurane experiments, a gaseous mixture of air and the indicated percentage of isoflurane was generated by a commercial vaporizer (Isotec 4, Surgivet) and was applied through a mask with a custom-built non-rebreathing circuit and a flow-rate of 3–4 l/min. Mice were induced at 4% isoflurane for ~30 s and then mounted on the microscope stage. The isoflurane concentration was then immediately reduced to 1% while the setup was prepared for subsequent imaging. After a time period of ~10 min, the isoflurane concentration was further reduced to 0.5% which was followed by a waiting interval of 15 min before image acquisition began. For ketamine/xylazine experiments, an initial dose of 65 mg/kg ketamine and 14 mg/kg xylazine was applied by intraperitoneal injection before mounting the animal on the stage. To maintain the anesthetic depth, 40% of the initial dose was injected 30–45 min after induction. When only ketamine was used, animals were injected with an initial dose of 130 mg/kg. To induce and maintain deep anesthesia, subsequent doses of 50 mg/kg were given to animals. For urethane experiments, animals were given a dose of 1.5 g/kg by intraperitoneal injection. Two supplemental doses of 0.7 g/kg were given 1 and 2 h after induction.

Using the imaging and anesthesia paradigms described above, we performed repeated two-photon activity imaging on the same mice under different anesthetic conditions. Sessions with different anesthetics were carried out in the following order: (1) ketamine/xylazine, (2) isoflurane, (3) ketamine only, (4) isoflurane for sound only trials, and finally (5) urethane. After each session with ketamine or ketamine/xylazine, animals were given at least 5 days to recover. In the case of isoflurane, they were given 2 days to recover. After completion of the urethane experiments, animals were sacrificed for immunofluorescence analysis.

### Immunofluorescence and confocal microscopy

**Figure 1:**
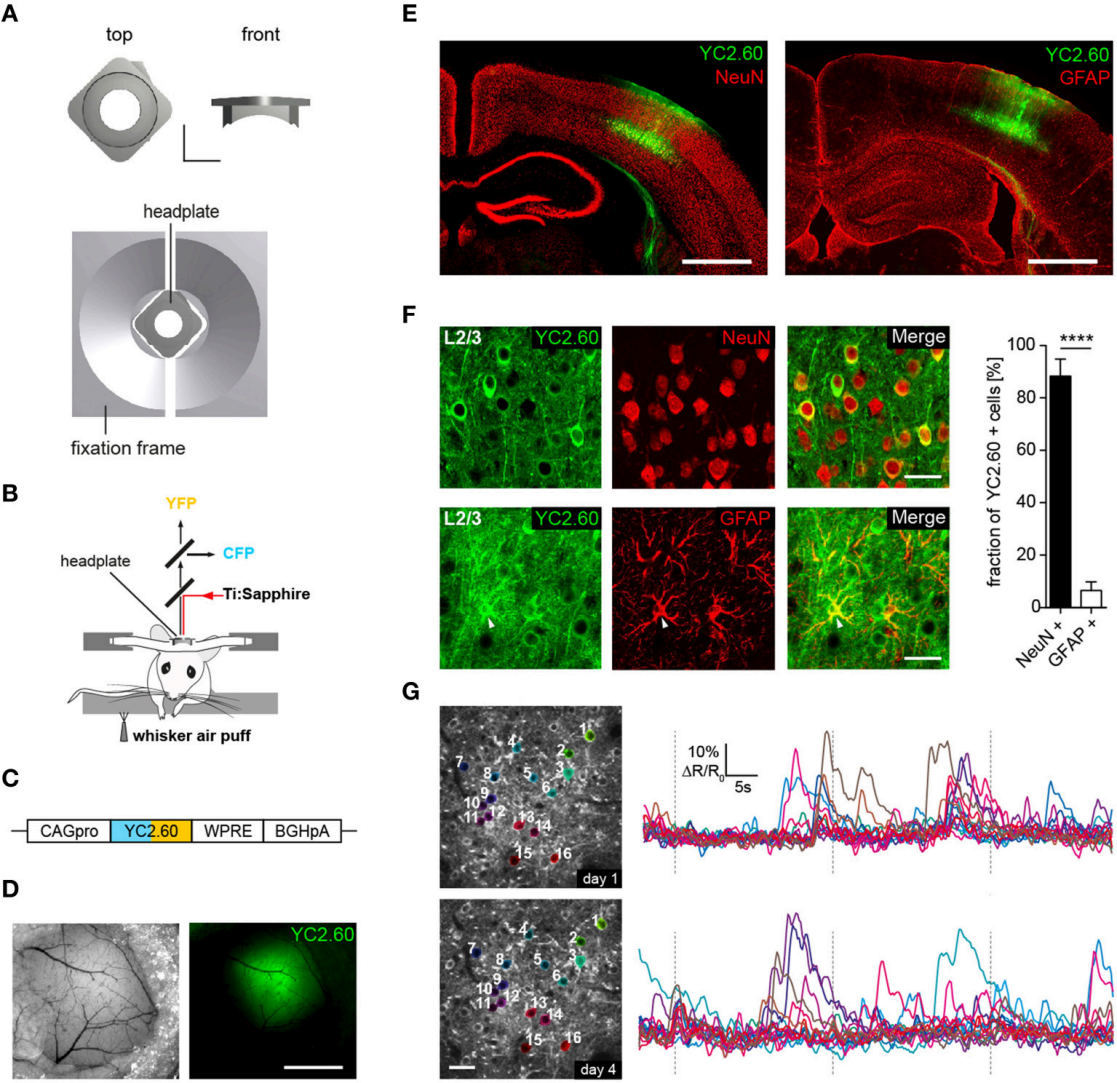
**Chronic ***in vivo*** two-photon activity imaging. (A, Top)** Titanium head-plate in rectangular design is shown with a top and a front view. Scale bar: 5 mm. **(Bottom)** Headplate in the corresponding fixation device. **(B)** Schematics for experimental setup for two-photon Ca^2+^ imaging of neural activity evoked by sensory stimulation. Sensory stimulation was achieved through an air-puff to the mystacial whiskers on the right side. **(C)** YC2.60 is expressed under control of the CAG promoter (CAGpro) and delivered into the barrel cortex by rAAV injection. WPRE, woodchuck-hepatitis posttranscriptional regulatory element; BGHpA, bovine growth hormone polyadenylation signal. **(D)** Wide-field images showing superficial blood vessels used for orientation and virus injected region within the barrel cortex. Scale bar: 1 mm. **(E)** Fluorescence images of brain slices from animals that were used for 2P-imaging show YC2.60 fluorescence in cortical layers. Note the absence of gliosis in the virus-injected region. Scale bar: 1 mm. **(F, Left)** Example images for immunofluorescence analysis for co-expression of YC2.60 and NeuN or GFAP in layer 2/3. Arrow indicates YC2.60-expressing glial cell. Scale bar: 30 μm. **(Right)** Quantification of co-expression of YC2.60 and NeuN or GFAP reveals strong expression tropism for neurons (90 ± 6%) over glial cells (6 ± 3%). Error bars = SEM, ^****^p < 10^−6^; unpaired Student's *t*-test, *n* = 6 mice (600 cells in total). **(G)** Chronic *in vivo* Ca^2+^ imaging in layer 2/3 of the barrel cortex. **(Left)** Images depict example brain region for functional imaging on day 1 and day 4 (depth = ~120 μm from the dura mater); scale bar: 25 μm. **(Right)** Example activity traces from all cells for a single trial on two different imaging days are shown.

Mice were anesthetized with either isoflurane or urethane, transcardially perfused with 1 × phosphate buffer saline (PBS) followed by 4% paraformaldehyde (PFA) in PBS, and brains were post-fixed for 12 h at 4°C. Free-floating sections (70 μm) were cut using a vibratome (Leica) and incubated for 1 h at room temperature in blocking solution (2% gelatin from cold water fish skin, 2% BSA and 0.1 % Triton-100 in PBS). Next, slices were incubated overnight with primary antibodies diluted in blocking solution at room temperature (RT). The following primary antibodies were used: NeuN (Millipore, mouse, 1:800 or 1:1000), GFAP (DAKO, rabbit; 1:600). Slices were either incubated with both primary antibodies simultaneously or different, adjacent slices were incubated with a single antibody (either NeuN or GFAP). No difference was found between the single- and double-staining. After washing with 1:3 diluted blocking solution, slices were placed in a solution containing a fluorophore-conjugated secondary antibody (goat-anti-mouse Cy3 and/or goat-anti-rabbit Cy5, Jackson Immuno Research Lab, 1:700 or 1:800, in 1:3 dilution of initial blocking solution) for 1.5 h at RT. Finally, slices were washed with PBS and mounted on glass slides using 80% glycerol in PBS. Wide-field fluorescence images were acquired with an Axioplan-2 microscope (Zeiss). Confocal microscope images were acquired at a resolution of 1024 × 1024 pixels with a Leica SP2, equipped with a 63 × glycerol immersion objective. For YC2.60 expression analysis, merged confocal images were analyzed manually for the co-localization of NeuN or GFAP with YC2.60. For a better print quality and red-to-black contrast perception, example NeuN and GFAP images in Figure 1 were enhanced slightly without altering any information content of the image.

#### Extraction of raw traces

All data was analyzed using custom Matlab (Mathworks) routines. Stack data was pre-processed to compensate for intra- and inter-trial shifts in the field of view. For this, the raw 128 × 128 pixel images from one of the channels of the YC2.60 stack data were fed into an ECC image alignment algorithm (Evangelidis and Psarakis, 2008) with the first image of each stack serving as an alignment template. This way, whole frame-to-frame x-y shifts could be successfully removed. Movements in the z-axis were ignored since out-of-focus movements did not lead to significant changes in ΔR/R. A 5% safety margin was taken into account on either side of the field of view and ROIs outside of this margin were removed from analysis. To ensure alignment to the first stack of each series of trials and therefore the defined ROI set, stacks were then aligned to their respective reference stack. This way, ROI sets were fully overlapping in between trials. Elliptical ROIs were defined manually and only clearly visible cells were chosen for analysis (Supplementary Figure 2). Once a ROI set was defined, it was maintained throughout all trials with only minor corrections applied to individual ROIs when necessary. Stacks whose inter-trial shifts were not successfully corrected were manually removed for further analysis.

Elliptical ROIs were further processed to confine analyzed pixels to actual cell boundaries and remove uncorrelated (neuropil) noise as described previously (Ozden et al., 2008). For this, ΔR/R traces of each pixel over time in each ROI were compared to each other using pairwise linear correlation coefficients (Pearson's r). For each ROI, pixels that exhibited correlation above a threshold, which was drawn from the population of all possible pairwise correlations in 10 overlapping time windows for every pixel trace and constituted more or equal to 10% of all pixels in the processed ROI, were selected for analysis. This ensured that ROIs (cells) that showed calcium events were optimally thresholded according to their activity signature and pixels (regions) that were not involved in the actual calcium event were neglected for further analyses. If the algorithm selected < 10% of pixels in a specific ROI, the respective ROI was not changed and all manually selected pixels were taken into account.

Mean *R*-values of each ROI were then extracted and further processed to yield ΔR/R traces for each cell. Briefly, raw *R*-values of each trace were fit with a generalized extreme value (GEV) distribution, which yielded a first estimate of R_0_. Given that calcium transients lead to positive deflection in a cell's activity trace, the baseline (R_0_) was then extracted as the mean of all values in between minimum (R) and GEV_mean_ + (GEV_mean_ − minimum(R)) to remove all large transients that would distort the baseline R_0_ and are apparent as outliers toward the right end of the distribution. This baseline was then used to calculate ΔR/R for each respective ROI. Due to this procedure, ΔR/R traces could be easily compared with each other since this analysis is robust toward large calcium transients that would otherwise shift R_0_ with them. To extract a peak threshold from ΔR/R traces, butterworth high-pass filtering at 0.1^*^frame rate cut-off was applied to remove all putative peaks and slow baseline drifts and traces were thresholded at >2.5 S.D. of a normal distribution parameter estimate. Traces were filtered using a digital smoothing polynomial filter (Savitzky–Golay) and the same parameters were used throughout the analysis. Peak times and amplitudes were extracted using the filtered traces that were robust against noisy peaks that would distort peak amplitudes and times. Peak amplitudes were further corrected by subtracting the minimum in 10 frames preceding the peak maximum to compensate for drifts (corrected amplitudes). Peaks that had a distance of < 10 frames to the last peak and had a corrected amplitude of < 1.5 S.D. of noise level were disregarded as noise/complex peaks. Traces and peaks were visually inspected after the analysis process was completed on a random subset of the data to find an optimal set of parameters, which was then used for all trials in all mice and days. If not otherwise denoted, peak amplitudes and number of events are given as mean over all trials per mouse/session.

#### Correlation analysis

We applied Pearson's pairwise correlation coefficient as a proxy for synchrony in the imaged neuronal network. All analyzed traces had a length of 300 frames (~74 s) and peak time points extracted from these traces were fed into Matlab's built-in “corr” function to extract Pearson's *r*. The accuracy of this calculation and the magnitude of correlation depend strongly on the correct peak time extraction. Peak times could be spread over adjacent frames in time because of the delayed registration due to the slow scan time of the imaging setup (~4 Hz) and therefore we corrected peak times according to the cell's relative y-position (top to bottom) in the field of view. This means that peaks get assigned a probability (*p*) value close to 1 if the cell's y-position comes close to the end of the scan path and close to 0 at the beginning of the scan path. This way, absolute peak times that are originally registered in only one frame are spread over two adjacent frames with the preceding frame getting an assigned peak probability of 1-p respectively (Ozden et al., 2008). Applying this method allows for a partial compensation of timing artifacts in peak registration.

**Figure 2:**
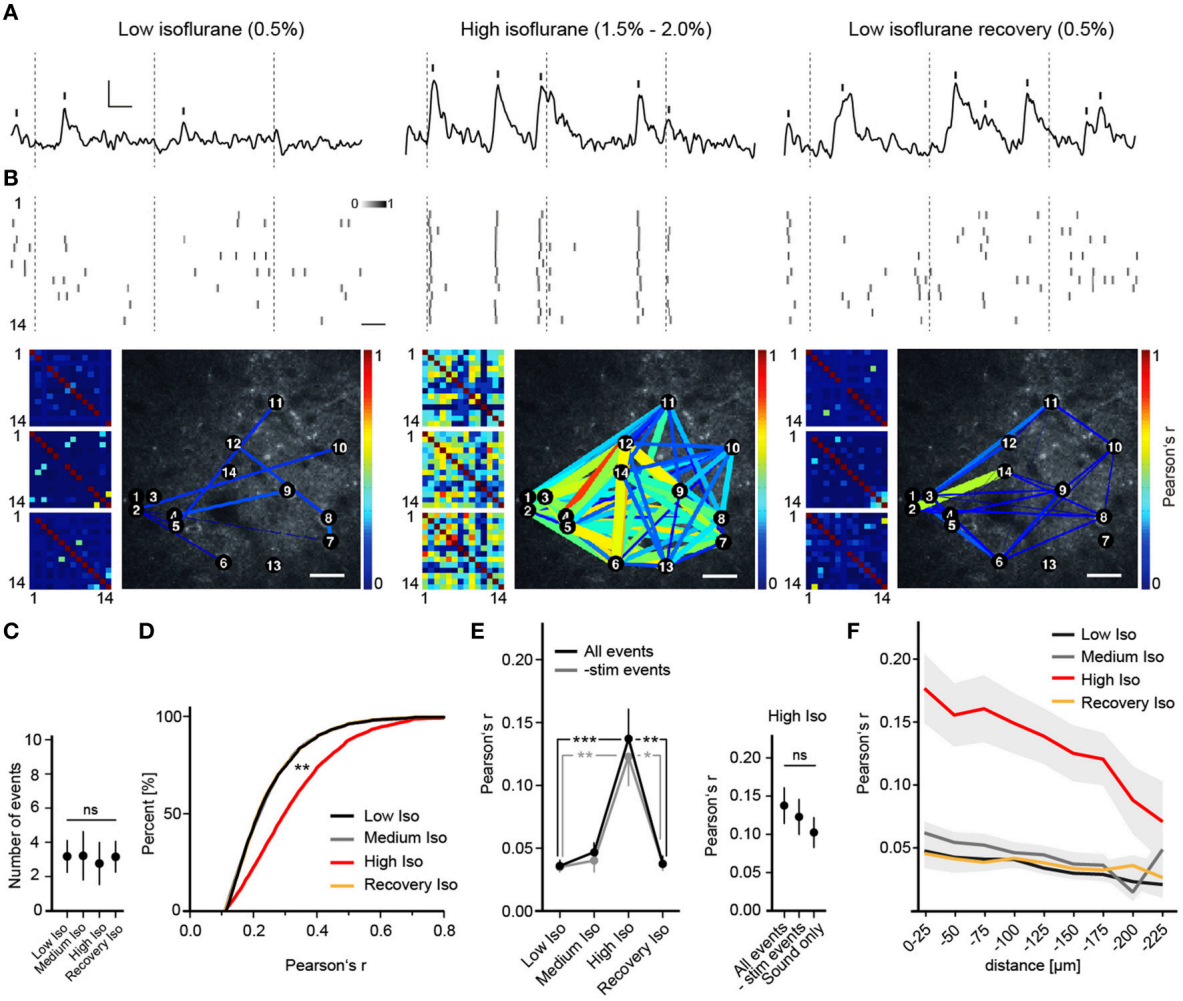
**Deep anesthesia enhances neural synchrony. (A)** Random example fluorescence traces of the same cell under different anesthetic conditions. Vertical bars mark detected peaks, inset: 5% ΔR/R (y-axis), 5 s (x-axis). **(B)** Extracted peak time points of all cells shown as probabilities over adjacent frames (**Top** panel, scale bar: 5 s), color-coded correlation matrices for Pearson's r of 3 example trials (**Bottom** panel, small pictures) and example correlation maps (**Bottom** panel, big pictures, scale bar: 30 μm). Inset: color-code for peak probability (white = 0 to black = 1). Dotted lines indicate time points of whisker stimulation. Correlation is expressed as Pearson's correlation coefficient (r) and color-coded from *r* = 0 (blue, no correlation) to *r* = 1 (red, perfect positive correlation). **(C)** The number of detected peaks per cell (mean over all trials and cells ± SD; ANOVA, *p* = 0.71). **(D)** Correlation r's filtered for significance (*p* < 0.05) shift toward higher values with high isoflurane: cumulative frequency distribution for all pairwise correlation r's (*p* < 0.01 High Iso vs. Low Iso). **(E)** High isoflurane concentrations induce a marked increase in overall synchrony that is independent of sensory stimulation. **(Left)** Mean pairwise correlation of all cell pairs for the indicated isoflurane concentration over all trials per mouse (mean ± SEM, ^*^p < 0.05, ^**^p < 0.01 ^***^p < 0.001). **(Right)** Summary of correlation *r*-values under a high isoflurane concentration (mean ± SEM). The same analysis as above was additionally performed for trials where only auditory stimulation was present (All peaks vs. subtracted *p* = 0.58, All peaks vs. Sound only; *p* = 0.43). **(F)** High isoflurane concentrations preferentially increase synchrony between nearby neurons. Shown is the mean pairwise correlation (Pearson's r) plotted against pairwise distance (bin size: 25 μm) (mean ± SEM).

**Figure 3:**
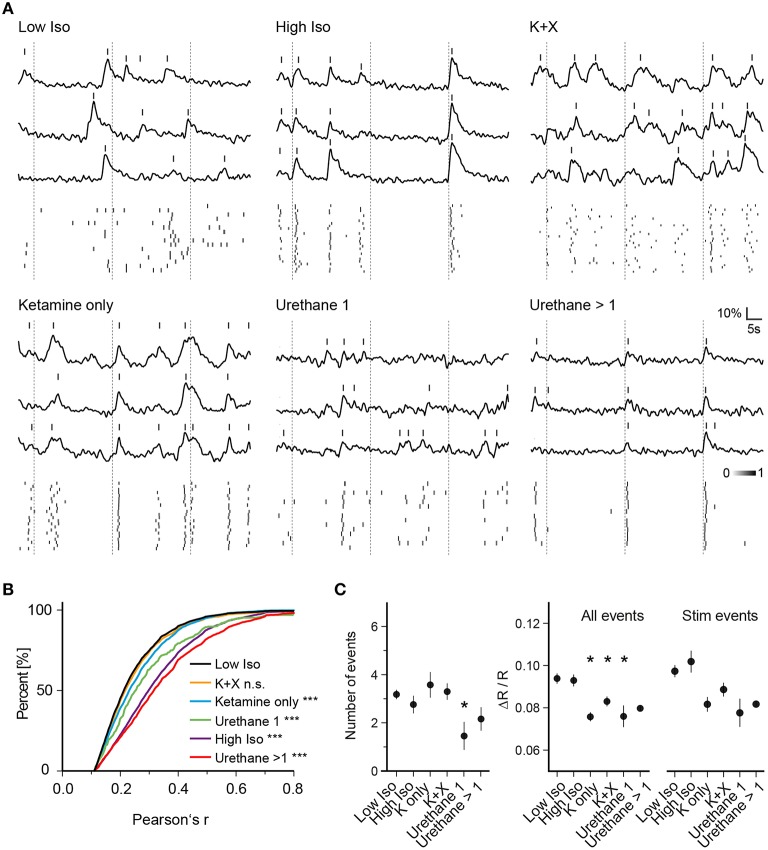
**Anesthesia-dependent synchrony is similarly induced by chemically distinct anesthetics. (A)** Example traces of three cells for one trial of each anesthetic condition are shown. **(Top)** Ca^2+^ signal traces; **(Bottom)** peak probability matrix. Dotted lines: time of air-puff stimulus **(B)** Cumulative frequency distributions of Pearson's r over all cell pairs and trials. Values were pre-filtered for significance level of *p* < 0.05 as in Figure 2D (see Section Materials and Methods for details); the low isoflurane dataset is shown in black and is taken as baseline condition and as a reference for statistical testing. A substantial rise in correlation between cell pairs was observed for all anesthetic conditions except Ketamine + Xylazine (K + X) (^***^p < 0.001 for all datasets vs. Low Iso except K + X). **(C)** Mean number of events per trial, mean amplitude of all events per trial and mean amplitude of all stimulus evoked responses per trial over all trials are plotted (mean ± SEM, ^*^p < 0.05).

To investigate the relation between distance and synchrony within cell pairs, the analysis was performed on distinct bins representing pairwise distance between cells from 0 to the maximum distance in micrometer. For the analysis represented in Figures 2E,F, the data was taken as is without any filtering applied after calculation of pairwise correlations. Figure 2D shows a cumulative frequency distribution of all pairwise correlations that were analyzed, irrespective of mouse or trial, but filtered for a significance level < 5% with built-in Matlab functions, since a high level of near-zero “noise” in pairwise correlations could have affected the analysis and pulled the mean correlations down. This analysis yielded highly similar results to those represented in Figure 2F. This approach was also chosen to process correlation measures taken for the datasets presented in Figure 3B.

#### Stimulus response analysis

**Figure 4:**
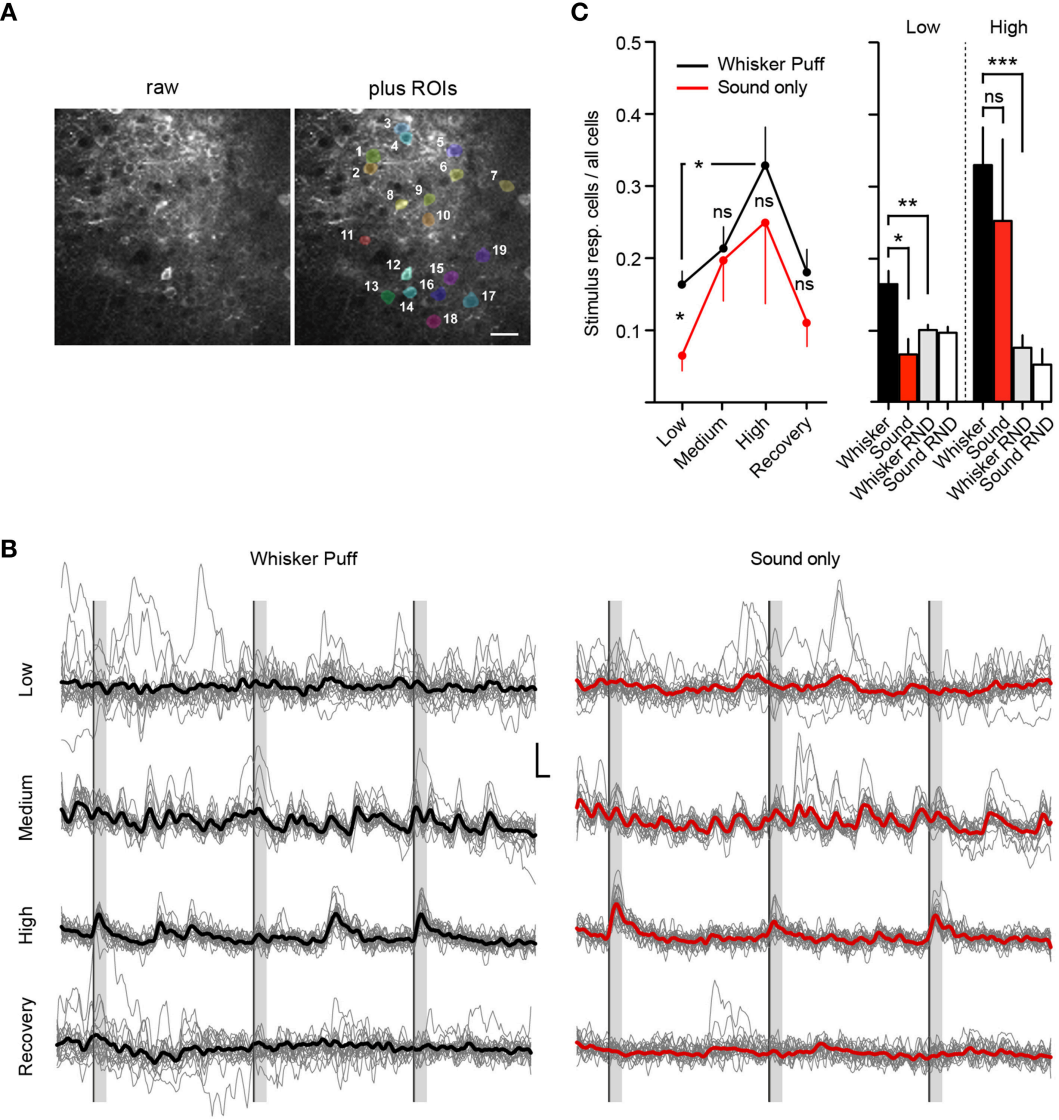
**Deep isoflurane anesthesia affects cortical information processing. (A)** Example field of view showing the raw image **(Left)** and an overlay of analyzed ROIs **(Right)**, scale bar 25 μm. **(B)** Example traces of imaged cells shown in **(A)** for whisker puff and sound only trials for each isoflurane concentration, inset: 10% ΔR/R (y-axis), 2 s (x-axis), thick lines indicate group average. **(C)** Fraction of neurons responding to whisker stimulation by an air-puff and sound-only stimulation under low (0.5%), medium (1.0%), and high (1.5–2.0%) isoflurane doses, and after recovery (0.5%) (Mean ± SEM, ^*^p < 0.05, only significant differences between stimulation groups and isoflurane treatments are marked in the figure). The differences in sensory responsiveness at low isoflurane vanished when the stimulus detection windows were randomized within datasets (RND) (see Section Materials and Methods) (mean ± SEM, ^**^p < 0.01, ^***^p < 0.001).

For the “-stim events” datasets presented in Figure 2E and the datasets in Figure 3C, stimulus related events were defined as calcium peaks following the stimulus (whisker air-puff/tone) time points in each trial within a 2 s time window. The rational behind this approach came from visual inspection of raw traces, observed stimulus responses during the experiments and the fact that some complex and large calcium transients that were elicited by whisker stimuli had a long rise time that shifted peak times significantly away from the stimulus initiation and therefore justified this long registration time window. Peak times and not rise times were taken as basis for analysis since the initiation time of calcium traces could not be clearly identified due to baseline noise. As for the analysis presented in Figure 4C (RND datasets), the original dataset was analyzed in the same way with 3 non-overlapping stimulus windows that were randomly chosen for each analyzed trial. If the observed effects were purely random and/or depending on increased frequency of detected peaks per trial that would in turn increase the probability of peaks falling inside the 2-s windows, the observed trends would not be expected to vanish with random permutation of detection windows.

### Statistical analysis

All statistics were calculated in GraphPad Prism. Non-parametric test statistics were used when normal distribution of analysis results could not be proven. For a detailed account of n-numbers for each trial and analysis see Supplementary Table 2. All data in main figures and Supplementary figures represent means over all cells and all trials in one session. Different mice and imaging sessions on different days were taken as independent samples.

## Results

### Experimental setup for chronic *in vivo* imaging of neuronal activity

We developed two custom-designed lightweight titanium head-plates for reliable fixation of mice for acute and chronic *in vivo* two-photon imaging (Figure 1A and Supplementary Figure 1). The head-plates are mounted on top of a cranial window that was implanted as previously described (Margolis et al., 2012; Figure 1B). As a reporter of neuronal activity, we selected the ratiometric Ca^2+^ indicator YC2.60, because of its high signal-to-noise ratio (SNR = 4.3 for single action potential detection) (Yamada et al., 2011), with rise and decay times of 185 ms/10 APs and 2.31 s/10 APs as measured in layer 2/3 cortical neurons *in vitro* (Yamada et al., 2011). YC2.60 is thus ideally suited to detect sparse activity patterns in layer 2/3 somatosensory cortex *in vivo* (Hendel et al., 2008; Yamada et al., 2011). For cell type specific gene delivery, we generated a recombinant adeno-associated virus (rAAV) equipped with a CAG promoter driving the expression of YC2.60 (rAAV-CAG-YC2.60) (Figure 1C). The virus (rAAV-CAG-YC2.60) was unilaterally injected into the barrel cortex of 6–7 weeks old C57BL/6N mice (Figures 1D,E). Immunofluorescence analysis showed that rAAV-CAG-YC2.60 provided expression predominantly in neuronal cells; only a small fraction of astrocytes were positive for YC2.60 expression. Cell type specific expression of YC2.60 was analyzed by co-labeling with anti-NeuN and anti-GFAP antibodies. In layer 2/3, 90 ± 6% (mean ± SEM) of YC2.60 expressing cells were neurons whereas only 6 ± 3% were glial cells (Figure 1F). Thus, our experimental approach permits reliable acute and chronic *in vivo* two-photon imaging of neuronal morphology and activity (Figure 1G and Supplementary Figure 3).

### Anesthesia causes a dose-dependent increase in neuronal synchrony

To investigate the effects of different anesthetics on stimulus-induced and spontaneous cortical neuronal activity, we performed repeated *in vivo* two-photon Ca^2+^ imaging sessions of approximately the same regions in layer 2/3 of the barrel cortex of the same mice under different anesthetic conditions (see Section Materials and Methods). We approximated the anesthetic state via a behavioral scoring system (see Supplementary Table 1). We found that under a low isoflurane concentration (0.5%), activity in neuronal populations was sparse and desynchronized (Figures 2A,B), and quite similar to that observed in the awake state (Greenberg et al., 2008). However, increasing isoflurane doses to 1.5–2.0% (High Isoflurane) caused marked changes in network dynamics; activity of nearly all neurons became highly synchronized (Figures 2A,B). This could not be attributed to a general increase in neural activity, which would increase correlation by simply augmenting the chance of simultaneous detection of Ca^2+^ events (Figure 2C, ANOVA, *p* = 0.68, *p* > 0.05 for Dunnett's multiple comparison test for all datasets vs. Low Iso). Interestingly, under 1.0% isoflurane (Medium Isoflurane), network dynamics were not significantly altered (Figures 2D–F), although the animal lost consciousness and transitioned into a deeper anesthetic state (see Section Materials and Methods and Supplementary Table 1). To quantify the observed synchronization between neuron pairs, we calculated Pearson's linear correlation coefficient (Pearson's r), which was significantly increased under 1.5–2% isoflurane (correlations were pre-filtered for a significance level of *p* < 0.05, see Section Materials and Methods for details, Figure 2D, ANOVA *p* = 0.0027, significance level of 0.01, Dunnett's multiple comparison test: *p* < 0.01 for High Iso vs. Low Iso). To determine if the network effects under high isoflurane concentrations are reversible, we switched back to 0.5% isoflurane (Recovery Isoflurane). Thirty minutes later, we found that values for pairwise correlation were similar to those observed under the initial low isoflurane dose (Figure 2D, 6143 ± 1220 cell pairs (mean ± SEM), Dunnett's multiple comparison test: *p* > 0.01 for Low Iso vs. Recovery Iso). These results indicate that the effects of isoflurane on cortical network activity are reversible within a short time period and add support for using isoflurane during surgical procedures and for the investigation of experience dependent cortical plasticity (Margolis et al., 2012). It is possible that the increased synchrony under a high isoflurane dose (1.5–2.0%) might be due to enhanced neural responses to sensory stimuli under anesthesia (Zandieh et al., 2003), which would lead to increased detection of simultaneous Ca^2+^ events within the same time windows. To address this issue, we subtracted all stimulus related peaks from the unfiltered data sets, and re-analyzed Pearson's r (-stim events, Figure 2E). The results for the -stim events data were almost identical to those described for the complete trial data (Figure 2E, mean number of trials: 11.32 SD ± 5, 7 mice. All events: Mann–Whitney, *p* = 0.0002; High Iso vs. Low Iso, *p* = 0.0033; High Iso vs. Recovery Iso. -stim events data: Mann–Whitney, *p* = 0.0017; High Iso vs. Low Iso, *p* = 0.0161; High Iso vs. Recovery Iso). We also included trials under a high isoflurane concentration in which the air-puff was turned off and only the air-puff trigger sound was active (see Section Materials and Methods for details). Under this condition as well, Pearson's r was not significantly different from the complete trial value (Figure 2E, right, Sound only). The trials with subtracted stimulus peaks and the ones with sound only stimulation showed no significant differences to the regular trial set (Figure 2E, mean number of trials: 9.58 ± 4 (mean ± SEM), 3 mice, All events vs. -stim events: Mann-Whitney, *p* = 0.58, All events vs. Sound only: Mann-Whitney, *p* = 0.43). Thus, the increased synchrony under a high isoflurane dose is not due to an enhancement of neural responses to sensory stimuli.

Next, we asked whether the pairwise correlation of two active neurons was dependent on their spatial distance. We calculated Pearson's r for discrete distance bins (Figure 2F) and found that neuronal activity was only correlated up to 200 μm between neurons, with higher distances (>200 μm) not showing significant differences in correlation compared to control (2-way ANOVA with Bonferroni multiple comparisons: Low Iso and Medium Iso vs. High Iso ^***^p < 0.001 for 0–175 μm, ^*^p < 0.05 for 175–200 μm, and *p* > 0.05 for 200–225 μm, Recovery Iso vs. High Iso ^***^p < 0.001 for 0–175 μm, and *p* > 0.05 for >175 μm; effect of treatment: *F* = 111.29, DFn = 3, DFd = 433, *p* < 0.0001; effect of distance: *F* = 4.41, DFn = 8, DFd = 433, *p* < 0.0001).

To determine whether the observed effects on neuronal network dynamics are specific to isoflurane or whether they represent a common feature of general anesthesia, we investigated the effects of ketamine, urethane, and ketamine/xylazine on activity patterns of neurons and compared those to the pattern recorded under low isoflurane (Figure 3). For ketamine, we intraperitoneally injected mice with an initial dose of 130 mg/kg body weight followed by another injection of 50 mg/kg 5 min before imaging. Similar to the high isoflurane concentration, pairwise correlation increased compared to the low isoflurane condition (Figures 3A,B, 3948 sampled cell pairs, ANOVA *p* < 0.0001, significance level of 0.01, Dunnett's multiple comparison test *p* < 0.001 for Ketamine only vs. Low Iso) although the magnitude of the effect was smaller than for high isoflurane concentrations. Interestingly, this effect was diminished when xylazine was included in the ketamine injection cocktail (Figures 3A,B, K + X, 4892 sampled cell pairs, Dunnett's multiple comparison test, *p* > 0.01).

Finally, we used urethane as another chemically distinct anesthetic. One urethane injection of 1.5 mg/kg body weight significantly increased Pearson's r (Figures 3A,B, Urethane 1, 360 sampled cell pairs, Dunnett's multiple comparison test, *p* < 0.001). After 1 and 2 h, mice were given an additional dose of urethane (0.7 g/kg) and the data from these two injections were pooled. These additional injections led to a further increase in pairwise correlations (Figures 3A,B, Urethane >1, 2621 sampled cell pairs, Dunnett's multiple comparison test, *p* < 0.001).

Next, we analyzed whether general anesthesia changed the number of detected events per trial compared to the low isoflurane (Low Iso) condition (Figure 3C, Number of events). Here, we only observed changes in trials of mice that received one urethane injection, with a significant reduction in number of detected peaks (ANOVA, *p* = 0.061, Dunnett's multiple comparison test *p* < 0.05 for Urethane 1 vs. Low Iso). When analyzing the average amplitudes of all Ca^2+^ transients (Figure 3C, All Events), we found a significant decrease for ketamine, ketamine/xylazine as well as for one urethane injection (ANOVA, *p* = 0.0003, Dunnett's multiple comparison test *p* < 0.05 for K only, K+X, and Urethane 1 vs. Low Iso). Although there was a trend toward lowered stimulus response amplitudes (Figure 3C, Stim Events) for these same conditions, statistical significance was not reached when compared to the Low Iso dataset (ANOVA, *p* = 0.028, Dunnett's multiple comparison test *p* > 0.05 for all datasets vs. Low Iso). Thus, deep anesthesia induced by high-dose isoflurane, ketamine, ketamine/xylazine, and multiple urethane treatments did not change the peak frequency or peak amplitude of stimulus induced Ca^2+^ transients in layer 2/3 neurons of the somatosensory cortex. However, deep anesthesia was accompanied by drastically enhanced synchronous Ca^2+^ activity patterns under all tested conditions except under ketamine/xylazine (K+X).

### Isoflurane anesthesia increases the fraction of stimulus responsive neurons and induces a breakdown in modality-specificity of stimulus responses

We next investigated how isoflurane anesthesia affects the number of whisker-evoked Ca^2+^ transients in layer 2/3 cortical neurons of the barrel cortex (Figure 4). We found that under a low isoflurane concentration (0.5%), which induced sedation, 17 ± 2% (Mean ± SEM) of cells responded to whisker stimulation by an air-puff (Figure 4C, Whisker Puff). However, under high isoflurane concentrations (1.5–2.0%), the fraction of neurons responding to whisker stimulation increased significantly to 33 ± 5% (Mean ± SEM) (*p* = 0.01, Unpaired *t*-test with Welch's correction). When animals were switched back to 0.5% isoflurane, the fraction of whisker-evoked responding cells returned back to 18 ± 3% (Mean ± SEM) (*p* = 0.03, Unpaired *t*-test with Welch's correction) indicating that deep anesthesia decreases the response specificity of somatosensory neurons by rendering previously unresponsive neurons sensitive to sensory stimuli.

We next investigated the specificity of neuronal responses to sensory modality in layer 2/3 barrel cortex by testing different concentrations of isoflurane for sound evoked responses in the barrel cortex (see Methods for details). Under a low isoflurane concentration (0.5%), there was a significant difference between the air puff-evoked neuronal responses [Figures 4B,C, Whisker Puff, 17 ± 2% (Mean ± SEM)] and those evoked by sound alone [Sound only, 7 ± 2% (Mean ± SEM)], which indicates that the barrel cortical neurons in layer 2/3 were highly responsive to whisker stimulation (and not sound) under low isoflurane (*p* = 0.015, unpaired *t*-test, Welch's correction). However, under high isoflurane concentrations (1.5–2.0%), this difference vanished, because the overall neuronal response probability to the sound stimulus increased steeply [25 ± 11% (Mean ± SEM), *p* = 0.6 for high isoflurane Whisker Puff vs. Sound only, unpaired *t*-test, Welch's correction]. Although there was a trend toward reversibility at 0.5% isoflurane, this did not reach statistical significance, indicating that sensory responses during the recovery trials were still compromised (Recovery Iso, Whisker Puff vs. Sound only, *p* = 0.2). As a further control experiment, we analyzed the recorded data again, but this time randomized the whisker-stimulus and sound-stimulus detection time windows (Figure 4C, see Section Materials and Methods for details). At 0.5% isoflurane, there was a clear difference in the fraction of stimulus responsive cells between the real trials and the randomized (RND) trials (Whisker vs. Whisker RND at Low Iso: *n* = 22, *p* = 0.0062, unpaired *t*-test, Welch's correction), supporting the conclusion that the air-puff onto whiskers induced specific sensory responses in layer 2/3 barrel cortical neurons. At high isoflurane doses, the fraction of responsive cells for the randomized trials remained low (Whisker RND vs. Whisker at High Iso: *n* = 12, *p* = 0.0005, unpaired *t*-test, Welch's correction), indicating that the heightened sensory responses were real and not an artifact generated by a higher number of spontaneous peaks occurring during the stimulus detection interval. Although the same trend was visible for the sound only dataset at high isoflurane (Sound only vs. Sound RND), these results did not reach significance (*p* = 0.23, unpaired *t*-test, Welch's correction). Our results therefore further support the hypothesis that deep isoflurane anesthesia induces a broadening of neuronal responses to tactile stimuli in layer 2/3 of the barrel cortex and increases responsiveness of these neurons to auditory stimuli.

## Discussion

Here we demonstrated the usefulness of *in vivo* two-photon imaging with the GECI YC2.60 for studying the effects of general anesthetics on visually identified neurons in the barrel cortex of mice. Previous studies used either large scale approaches such as surface EEG recordings (Supp et al., 2011) or smaller scale methods of unidentified neurons such as electrophysiological unit recordings (Erchova et al., 2002) to investigate similar phenomena. We fill this gap by measuring the effects of anesthesia on up to 30 visually identified barrel cortex neurons simultaneously. Our high-resolution imaging paradigm holds promise for future studies as it allows the detailed dissection of the effects of anesthesia on different cell types, both excitatory and inhibitory neurons (Taub et al., 2013), as well as astrocytes (Thrane et al., 2012). It is known that general anesthetics can provoke short- and long-lasting effects (Perouansky and Hemmings, 2009), including dementia (Kapila et al., 2014). Chronic *in vivo* two-photon imaging as used in our study will enable the monitoring of the same cells over a period of hours on the same day and over many days (Margolis et al., 2012). In addition, the inducible gene deletion technology using AAV to delete specific target genes in selective brain region(s) (Hasan et al., 2013; Dogbevia et al., 2015) will help to link the role of gene function to changes in circuit dynamics induced by general anesthetics in individual cells and entire neuronal networks over time.

Here, we have shown that different general anesthetics (isoflurane, ketamine and urethane) induce local neuronal synchrony in layer 2/3 of the somatosensory cortex similar to results of Ca^2+^ imaging studies in the visual cortex of mice (Goltstein et al., 2015). In line with previous findings (Erchova et al., 2002) the observed correlation in neuronal activity of layer 2/3 neurons in the cortex under anesthesia was highest between nearby neurons. Synchronized network activity under anesthesia in the form of burst suppression (BS) patterns in EEG recordings has been studied in various animal models. While BS patterns have been examined extensively *in vitro* as well as *in vivo* using electrophysiological methods and modeling approaches (Ching et al., 2012; Bojak et al., 2015), the physiological and cellular mechanisms underlying their development are still not well understood (Bojak et al., 2015). We propose that our methodology, combined with genetic targeting of specific neuronal subtypes will enable the detailed interrogation of network mechanisms that lead to the emergence and after-effects of these patterns over different time scales (sub-second to weeks). *In vitro* studies in cortical slices and *in vivo* studies in rodents and cats using thalamic lesions suggest that such activity patterns might be generated in the cortex (Steriade et al., 1993; Sanchez-Vives and McCormick, 2000; Constantinople and Bruno, 2011; Stroh et al., 2013), possibly by pyramidal neurons in infra-granular cortical layers (Stroh et al., 2013; Sitdikova et al., 2014). Although pharmacological studies suggest the dependence of the observed activity patterns on specific receptor activity (Steriade et al., 1994; Lukatch et al., 2005; Kroeger and Amzica, 2007), it is still unclear which cell types and specific mechanism(s) are involved in the emergence of these activity patterns. Moreover, GABA_*A*_R agonists (Lukatch et al., 2005) as well as suppression of inhibition (Ferron et al., 2009) produced similar activity patterns in response to anesthetic agents. We propose that cellular resolution 2P *in vivo* Ca^2+^ imaging together with the ability to target specific cell types via GECIs such as YC2.60 can greatly advance our understanding of the emergence of BS and slow wave patterns in cortical networks under anesthesia and in other conditions like sleep and coma.

Furthermore, the role of synchrony in conscious perception has long been debated, but it is important to distinguish between different spatial scales of synchrony, ranging from correlated activity of different brain regions on one side to synchrony in highly localized neuronal networks on the other side. It has been proposed that certain forms of long-range synchronization between different brain regions might bind various sensory responses to a coherent perceptual experience (Engel and Singer, 2001). It was also proposed that certain forms of excessive interregional neuronal synchronization under anesthesia disrupt meaningful information processing and to induce unconsciousness (Supp et al., 2011). Our results provide further evidence for the commonness of local, short-range synchrony during most anesthetic treatments (Erchova et al., 2002). However, we also note that animals showed decreased spontaneous movements under certain anesthetic treatments, which did not induce neural synchrony (i.e., ketamine/xylazine and 1% isoflurane). This indicates that the increase in correlation observed in our experiments and previous studies might not be necessary for the loss of consciousness.

One caveat is that different anesthetics in our study might have influenced Ca^2+^ signaling and thus GECI signals that are independent of neuronal spiking activity. However, a previous study has shown that Ca^2+^ transients measured by GECIs correlate to action potentials under deep anesthesia (Lütcke et al., 2010). We also think it is unlikely that the correlated Ca^2+^ transients under deep anesthesia are caused by simple changes in Ca^2+^ signaling efficiency, as the transients retain the characteristic shape of fast rise and slow-exponential decay times indicative of Ca^2+^ transients caused by neuronal spiking. The considerations on Ca^2+^ signaling efficiency might be most relevant to evaluation of transient amplitudes in Figure 3. However, our Ca^2+^ imaging data is in line with previous findings that used purely electrophysiological recordings, which further supports that changes in Ca^2+^signals in our study are due to changes in neuronal spiking.

In addition to its effects on synchrony, we found that high dose isoflurane profoundly increased the fraction of whisker-responsive neurons in layer 2/3 of the somatosensory cortex, possibly rendering previously unresponsive neurons sensitive to sensory stimuli. Moreover, we show that this process is reversible, since lowering the isoflurane concentration decreased the fraction of whisker responsive cells back to initial values. It is unclear which network mechanisms underlie the observed effects. One possible explanation is that the altered network response is caused by a breakdown of local inhibition, which would normally confine the neuronal responses to individual neurons, thereby favoring sparse firing patterns over the synchronized responses in local neuronal networks. We cannot exclude, however, that the anesthesia-induced changes in network wide synchrony are caused by a downstream network. Indeed our results bear similarity to the broadening of thalamic receptive fields under anesthesia (Friedberg et al., 1999).

The above results are also interesting in light of previous work reporting a contraction of cortical whisker fields under urethane anesthesia (Erchova et al., 2002). This report found that barrel cortex neurons become less responsive to whisker deflections in adjacent barrels which was interpreted as a decrease in overall barrel cortex responsiveness thereby contradicting our results. However, one discrepancy lies in the anesthetic used. Here, we use isoflurane while Erchova et al. used urethane which might explain differences in experimental observations because different anesthetics, although both enhancing neuronal synchrony, might affect sensory responses in distinct manners. Also, our field of view is smaller (max. 200 μm) than the cell pairs recorded in the aforementioned study (400 μm interelectrode distance) and it might be that neurons in the immediate vicinity of the barrel center might become more responsive while neurons further away become less responsive (i.e., the whisker territory retracts, but neurons within the now smaller area become more responsive to sensory stimuli). Also, in the previous report, recordings were performed in infragranular layers whereas we record in supragranular layer 2/3 and it might thus be possible that different neuronal subtypes respond differently to anesthetic treatment. In support of our findings of increased and unspecific neuronal responses, a previous report has shown that neurons in adjacent barrel columns become more responsive under urethane anesthesia (Simons et al., 1992).

Not only did whisker-evoked neuronal responses in the layer 2/3 barrel cortex in our study become more diffuse under anesthesia, but neurons also responded to a tone stimulus. Similar to our observation of sound-evoked responses in the barrel cortex, previous studies reported sensory stimulus evoked neuronal responses under deep anesthesia in cortical areas other than those directly linked to the processing of the specific stimulus quality (Land et al., 2012). Together these results provide strong evidence for the distortion of dedicated sensory boundaries in the cortex under anesthesia. In contrast to the fraction of layer 2/3 neurons that responded to whisker stimulation, tone-induced neuronal responses in the barrel cortex were not completely reversible within a recovery period of about 1 h, which hints toward a slow reconstitution of these boundaries after deep anesthesia.

It is tempting to speculate that a breakdown in the multimodal sensory signal transduction in the anesthetized cortex is physiologically related to the pathophysiological state as described in synesthesia, a condition where stimulation of one sense leads to an involuntary and simultaneous perception within another sense, for example, auditory stimulus evoked visual responses (Harvey, 2013). Anesthesia and synesthesia might share certain cellular mechanisms, such as increased synchrony (Harvey, 2013) or modulation of inhibitory neural activity (Terhune et al., 2014) that lead to a breakdown of sensory boundaries. Therefore, two-photon Ca^2+^ imaging of sensory circuits under anesthesia might also provide valuable insights into the pathophysiology of synesthesia.

Notably, in the barrel cortex, astrocytic activity response shows the opposite effect in deeply anesthetized mice to those reported here. Synchronous activity of the glia network is disrupted under deep anesthesia and astrocytic Ca^2+^ responses are reduced (Thrane et al., 2012). Considering the delayed, but well defined timing of the glia response to whisker induced activity (Wang et al., 2006; Thrane et al., 2012), it seems quite unlikely that the glia network is simply responding to local neuronal action potentials. It is possible that the astrocytes are integrating and responding to multiple local and global neuronal activities. However, currently we cannot exclude that the described glial responses to different anesthetic drugs may constitute a non-neuronal mechanism for sedation and unconsciousness.

We propose that our findings can guide the selection of anesthetic treatments for future studies. We noted that an isoflurane dose of 1.0% (which completely immobilized the animal) did not change network parameters compared to the sedated state (0.5% isoflurane) and might therefore be suited for studies in which mice have to be immobilized during the investigation of brain activity. Taken together with the quick reversibility of its effects on neuronal activity as shown in our study, we therefore recommend low dose isoflurane (0.5–1.0%) as the anesthetic of choice for the investigation of neuronal sensory processing in living mice.

In conclusion, we propose that *in vivo* two-photon Ca^2+^ imaging of animals under different anesthetic conditions is an attractive method for dissecting molecular and cellular mechanisms surrounding anesthetic action and consciousness. Studies examining anesthetic effects on neural networks can provide important insight into the operation of circuit function when consciousness is lost and regained. To identify molecular targets and signaling pathways orchestrating the effects of general anesthesia, it will be imperative to apply advanced genetic tools for manipulating candidate gene(s) in local and distributed brain circuits for future studies (Hasan et al., 2013; Dogbevia et al., 2015). Optogenetic manipulation of specific neuron classes in the cortex and/or projections from other brain regions during two-photon activity imaging will further help to dissect cellular and circuit dynamics of general anesthesia.

## Author contributions

TL and MH designed the experiments. TL performed chronic *in vivo* two-photon imaging under different anesthetic conditions and helped with data analysis. DD performed rAAV injections and chronic window surgeries on mice. HO wrote analyses routines and performed statistical analysis. TN and AM provided YC2.60. RS provided reagents. MH supervised the project. TL, HO, RS, and MH wrote the paper.

### Conflict of interest statement

The authors declare that the research was conducted in the absence of any commercial or financial relationships that could be construed as a potential conflict of interest.
