# Supplementary material for: General Anesthetic Conditions Induce Network Synchrony and Disrupt Sensory Processing in the Cortex
Source: Front Cell Neurosci. 2016 Apr 14;10:64. doi: 10.3389/fncel.2016.00064 (PMC4830828; doi:10.3389/fncel.2016.00064)
Supplement: Supplementary file 1 [file DataSheet1.PDF]

*Supplementary Material*

# General anesthetic conditions induce network synchrony and disrupt sensory boundaries in the cortex

Thomas Lissek[1,2$], Horst Andreas Obenhaus[1$], Désirée A. W. Ditzel[1], Takeharu Nagai[3], Atsushi Miyawaki[4], Rolf Sprengel[1], Mazahir T. Hasan[1,5]*

[1]Department of Molecular Neurobiology, Max Planck Institute for Medical Research, Heidelberg, Germany

[2]Department of Neurobiology, Interdisciplinary Center for Neurosciences, University of Heidelberg, Heidelberg, Germany

[3]Hokkaido University, Hokkaido, Japan

[4]RIKEN, Saitama, Japan

[5]NeuroCure, Charite-Universitätsmedizin, Berlin, Germany

$ Co-first author

* Correspondence: mazahir.hasan@charite.de

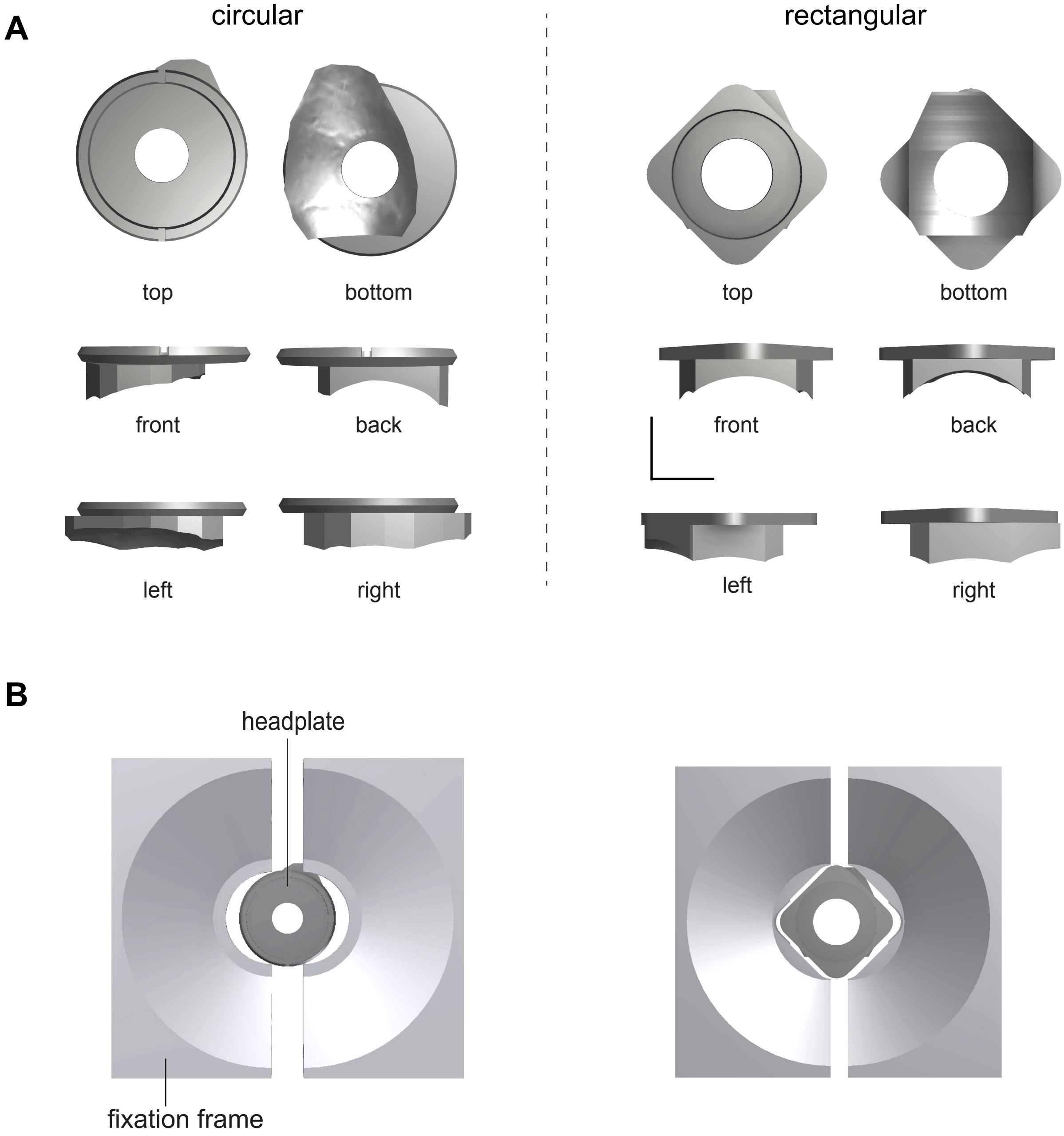


**Supplementary Figure 1 Titanium headplates for chronic in vivo calcium imaging in the barrel cortex.** (a) Two versions of titanium headplates for reproducible animal fixation under the microscope are shown from different angles. The circular design offers rotational flexibility around the vertical axis in case the animal needs to be rotated relative to the microscope stage. The two indents allow for orientation. The rectangular design allows maximum reproducibility of animal fixation and facilitates the revisiting of defined brain regions for chronic imaging. Scale bar: 5 mm (both axes). (b) Both headplate designs are shown in their corresponding fixation frames.

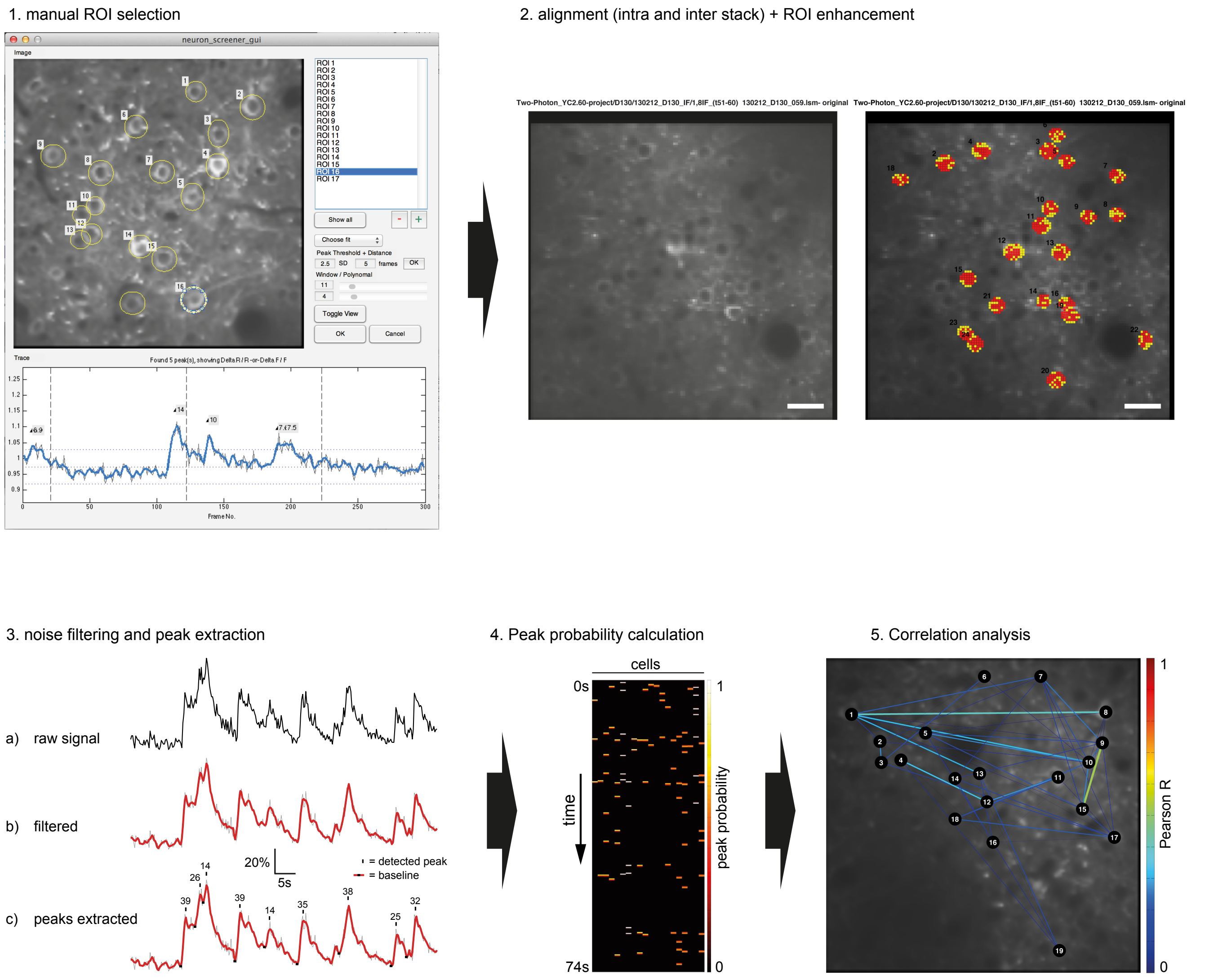


**Supplementary Figure 2 Workflow for image analysis.** For details on calculations, we refer to the methods section. First, ROIs are drawn manually and parameters for subsequent signal analysis are chosen in a custom graphical user interface (1). Image stacks were then automatically corrected for inter- and intratrial frameshifts (not shown). To enhance signal-to-noise ratio and to reduce neuropil signal contamination, ROIs were automatically refined by pixel fluorescence correlation analysis (2). The extracted raw signal was then noise-filtered and peaks were automatically extracted with individual baseline correction for each peak (3). To eliminate detection bias of raster scanning, each peak was assigned a probability value according to its position in the field of view (4). Peaks were then analyzed for pairwise correlation (5) and stimulus responsiveness (not shown).

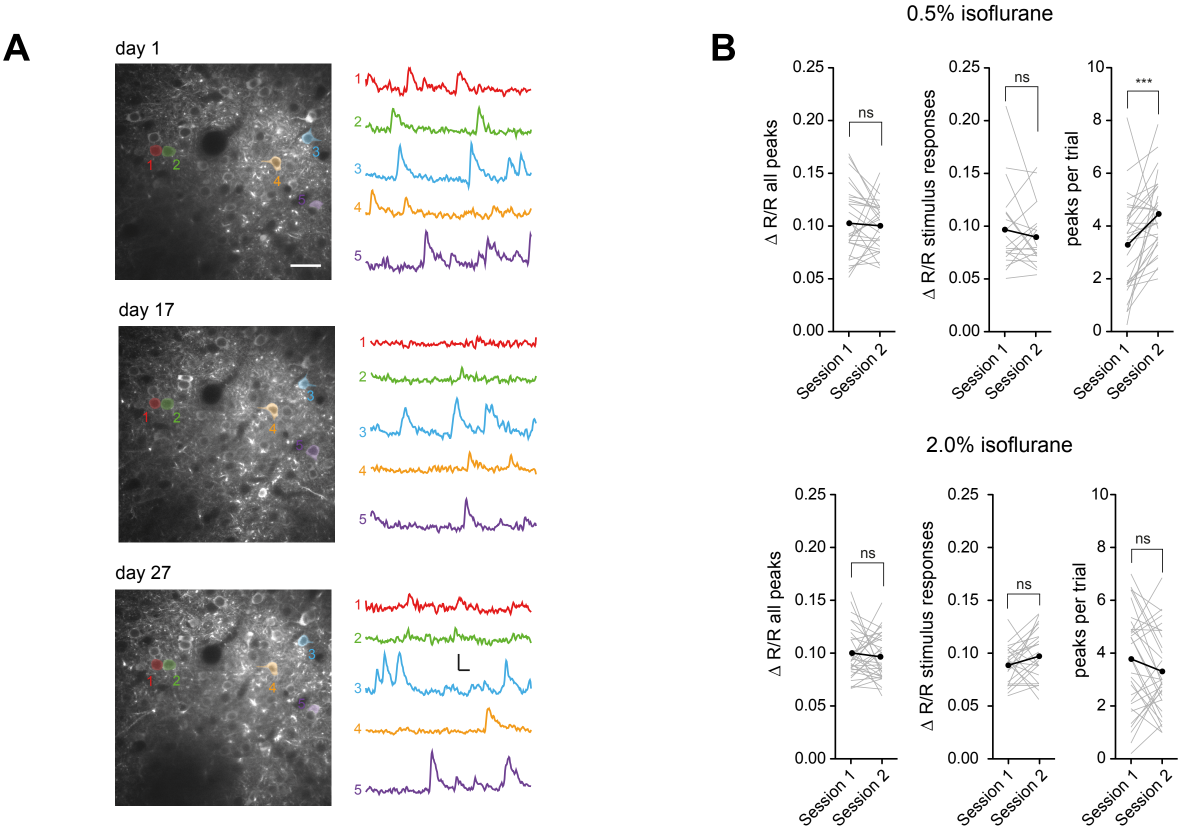


**Supplementary Figure 3 Chronic in vivo calcium imaging** (a) Chronic in vivo Ca2+ imaging in layer 2/3 of the barrel cortex. Images depict brain region for functional imaging on days 1, 17 and 27 (depth = ~120 µm from the dura mater). One example trace per cell and day is shown. All neurons showed regular activity on all experimental days. All activity shown was recorded under 0.5% isoflurane anesthesia. Scale bar: 30 µm. (b) Comparison of cells that were identified in two sessions during 0.5% and 2.0% Isoflurane anesthesia. Grey lines indicate single cells, black thick lines indicate group mean. Cells were compared for: ΔR/R for all peaks (including spontaneous and stimulus evoked events), ΔR/R of stimulus responses (stimulus evoked events only) and number of events per trial (peaks per trial). Activity parameters stayed mostly stable in between imaging sessions (27.8 ± 2 cells (Mean ± SEM), 4 mice, Wilcoxon matched-pairs signed rank test, $p>0.05$; $p = 0.0003$ for peak per trial Session 1 vs. Session 2 at 0.5% Isoflurane).

**Supplementary Table 1**

## Anesthesia states

- = absent; +/- = weakly present or absent; + = moderate; ++ = strong

**Isoflurane**

| Anesthetic depth | erect posture | spont. limb movements | spont. whisker movements | reaction to paw and tail pinch | breathing |
|---|---|---|---|---|---|
| awake | ++ | ++ | ++ | ++ | physiologic |
| light (sedation or recovery) | ++ | + | ++ | ++ | physiologic |
| medium | - | - | - | +/- | ~ 2 Hz |
| deep | - | - | - | - | ~ 0.5 Hz |

**Ketamine and Ketamine/Xylazine**

| | erect posture | spont. limb movements | spont. whisker movements | reaction to paw and tail pinch |
|---|---|---|---|---|
| light (recovery) | + | + | ++ | ++ |
| medium | - | - | + | +/- |
| deep | - | - | - | - |

**Urethane**
Under urethane anesthesia, all animals showed no reaction to paw pinch and no spontaneous whisker or limb movements. After the first two injections, the breathing frequency remained at the physiological level. After the third injection, the breathing frequency started to decrease continuously.

## Supplementary Table 2

| | Isoflurane correlation analysis | | | whisker | mouse n |
|---|---|---|---|---|---|
| | Low | Medium | High | Recovery | |
| **n independent** | 22 | 12 | 12 | 10 | **7** |
| Minimum | 5 | 9 | 5 | 4 | |
| Maximum | 34 | 20 | 20 | 22 | |
| Median | 10 | 11 | 11 | 9 | |
| Mean | 11.23 | 11.83 | 11.58 | 10.6 | |
| Std Dev | 5.887 | 3.433 | 4.963 | 5.719 | |

| | Isoflurane correlation analysis | | | sound only | |
|---|---|---|---|---|---|
| | Low | Medium | High | Recovery | |
| **n independent** | 3 | 3 | 3 | 3 | **3** |
| Minimum | 7 | 8 | 9 | 4 | |
| Maximum | 8 | 20 | 14 | 9 | |
| Median | 7 | 11 | 10 | 8 | |
| Mean | 7.333 | 13 | 11 | 7 | |
| Std Dev | 0.5774 | 6.245 | 2.646 | 2.646 | |

| | Ketamine + Xylazine | |
|---|---|---|
| | K+X | |
| **n independent** | 12 | **8** |
| Minimum | 14 | |
| Maximum | 52 | |
| Median | 25.5 | |
| Mean | 27.75 | |
| Std Dev | 12.29 | |

| | Ketamine only | |
|---|---|---|
| **n independent** | 3 | **3** |
| Minimum | 22 | |
| Maximum | 44 | |
| Median | 22 | |
| Mean | 29.33 | |
| Std Dev | 12.7 | |

| | Urethane | | |
|---|---|---|---|
| | 1 | >1 | |
| **n** | 3 | 3 | **3** |
| Minimum | 11 | 15 | |
| Maximum | 25 | 25 | |
| Median | 15 | 22 | |
| Mean | 17 | 20.67 | |
| Std Dev | 7.211 | 5.132 | |
